# Around the Hospitals

**Published:** 1893-12-16

**Authors:** 


### Around the Hospitals.

Notice to the Managers of Institutions.—Special space is now reserved for the insertion of notices of hospital meetings and festivals. The Editor requests that all notices may reach The Hospital Office, 428, Strand, at least one week before the date of each meeting.

**The Bridgwater Infirmary.**—The management of this institution have adopted a scheme for collecting subscriptions in the surrounding districts which, if fortunately managed, should help to strengthen the infirmary and increase interest in it. They have elected secretaries in each parish, who have undertaken to bring forward the wants of the infirmary in their localities, and obtain subscriptions. If this plan works well, as it should do, the yearly adverse balance on the accounts will be removed. The number of patients treated is increasing, and therefore diminution in the expenses is not to be expected. The Ladies' Association does good work by maintaining a bed at a convalescent home for the use of the infirmary patients. We cannot admire the manner in which the annual report of the infirmary is arranged. The concise and brief record of the year's work is so wedged in between lists of subscribers and donors as to be apt to be overlooked altogether.

**Moseley Hall Convalescent Home for Children.**—This convalescent home, which had its small beginnings in the establishment of a Samaritan fund by some ladies in connection with the Birmingham Children's Hospital, has now held its first annual meeting and presented its report for the same period. Moseley Hall represents part of a gift of £30,000 by Mr. Richard Cadbury for the establishment of a convalescent home for children. Twenty beds are retained for the use of the children's hospital, which previously possessed a little home at Arrowfield Top in connection with the Samaritan Fund alluded to before, and which is amalgamated with the larger scheme. During the past year 133 patients out of a total of 337 were received from the children's hospital. Most of the children stay a month, the cost for maintenance being about 3s. 6d. per week per head. A charge is made for residence, but neither the income derived from this nor the subscriptions yet are quite adequate to cover the expenses, and larger sums will be necessary to utilise the 100 available beds. Great interest is felt for the institution in the neighbourhood, and many are the presents received and entertainments given for the benefit of the little inmates. There can be no doubt as to the beneficial character of the work at Moseley Hall, and we feel sure that Mr. Richard Cadbury's munificence will be emulated, and the success of the first year's experience be still further increased as time goes on.

